# Teriparatide and Abaloparatide Have a Similar Effect on Bone in Mice

**DOI:** 10.3389/fendo.2021.628994

**Published:** 2021-04-19

**Authors:** Mikkel Bo Brent, Frederik Eriksen Stoltenborg, Annemarie Brüel, Jesper Skovhus Thomsen

**Affiliations:** Department of Biomedicine, Aarhus University, Aarhus, Denmark

**Keywords:** μCT, bone formation, PTH, bone strength, abaloparatide

## Abstract

Three bone anabolic pharmaceuticals are currently approved for treatment of osteoporosis, teriparatide (PTH (1–34)), the parathyroid hormone-related protein analog abaloparatide (ABL), and romosozumab. The present study compared the effect of intermittent PTH (1–34) and ABL on bone tissue directly mole-to-mole in female mice. Forty-seven C57BL/6 mice were randomly allocated to the following groups: Baseline (*n* = 11), Control (Ctrl) (*n* = 12), PTH (*n* = 12), and ABL (*n* = 12). The mice were injected s.c. with PTH (100 µg/kg), ABL (96 µg/kg), or saline (Ctrl) five days a week for three weeks. To assess the effect of PTH and ABL, the hindlimb bones were analyzed with DXA, µCT, mechanical testing, dynamic bone histomorphometry, and histological quantification of bone cells. In addition, serum calcium concentration was determined. PTH and ABL significantly increased femoral areal bone mineral density (aBMD) (borderline significant *p* = 0.06 for PTH), femoral mid-diaphyseal bone strength, femoral metaphyseal and epiphyseal and vertebral bone volume fraction (BV/TV), connectivity density, volumetric bone mineral density (vBMD), and bone formation rate (BFR/BS) compared to Ctrl. In addition, ABL also significantly increased mid-diaphyseal cortical thickness and bone area compared to Ctrl. Neither PTH nor ABL significantly increased bone strength at the femoral neck. In conclusion, abaloparatide and PTH have similar bone anabolic properties when compared directly mole-to-mole in mice.

## Introduction

Osteoporosis is a disease characterized by decreased bone mineral density, impaired trabecular microarchitecture, and increased risk of fracture (1). In 2015, an estimated 20 million people were suffering from osteoporosis in France, Germany, Italy, Spain, UK, and Sweden combined (2). The International Osteoporosis Foundation estimates that more than 61 million adults in the United States are affected by the disease in 2020. Osteoporosis is a global disease present in all regions of the world, and the prevalence is likely going to increase in the coming years due to aging populations and lifestyle changes (3). Several pharmaceutical interventions have been tested and approved for treatment of osteoporosis and prevention of the subsequent debilitating osteoporotic fractures (4). Until 2017, teriparatide was the only FDA (Food and Drug Administration) and EMA (European Medicines Agency) approved bone anabolic treatment for osteoporosis. However, in 2017 abaloparatide (ABL) was also approved by the FDA for treatment of postmenopausal osteoporosis in the United States.

Teriparatide is a shortened form of human parathyroid hormone (PTH), consisting of the first 34 amino acids of the hormone (5, 6). When PTH is administered intermittently, a high bone turnover with positive bone formation balance occurs resulting in increased bone mass (7). In contrast to intermittent administration of PTH, continuously elevated serum PTH, as seen in patients suffering from hyperparathyroidism, results in reduced osteogenesis and decreased bone mass (8). PTH interact with the parathyroid hormone 1 receptor (PTH1R) expressed on osteoblasts, osteocytes, bone lining cells, and other mesenchymal-derived cells (9, 10). Downstream, an intracellular signaling cascade is initiated through increased production of intracellular cAMP ultimately resulting in bone formation and to a lesser extend osteoclastic bone resorption (11–13). The PTH1R exits in two distinct receptor conformations, R^0^ and R^G^ (14). Interaction with the R^0^-conformation results in a more gradual and prolonged increase of cAMP compared to the rapid and more swift increase by interaction with the R^G^-conformation (15, 16). Teriparatide has higher affinity for the R^0^-conformation of the PTH1R, thus inducing a longer-lasting signaling response, but also a concomitant increased risk of hypercalcemia due to prolonged osteoclastic stimulation (15–17). The two different conformations of the PTH1R have been exploited to produce ABL, a parathyroid hormone-related protein (PTHrP) analog with higher affinity for the R^G^-conformation of PTH1R. The more rapid cAMP increase induced by ABL has been linked to lower osteoclastic bone resorption, and this may explain why patients treated with ABL are less likely to develop hypercalcemia compared to patients treated with teriparatide (18). However, the effects of teriparatide and ABL are complex, pervasive, and still not completely elucidated.

Only one randomized clinical trial has been conducted comparing the ability of teriparatide and ABL to counteract osteoporosis in postmenopausal women, and in that study, the doses of the two drugs were not the same (18). Similarly, only few studies have compared the bone anabolic effects of teriparatide directly to those of ABL, and the studies performed in laboratory animals have used different doses and study durations except for the study by Le Henaff et al. (19–21). That study investigated the effect of the same dose of teriparatide and ABL in mice, however, their treatment regimen did not take relative molecular mass of the two drugs into consideration. Importantly, the mechanical properties of the bones, including the clinically important femoral neck fracture strength, have not been reported in studies comparing treatment with PTH and ABL at comparable doses. In the present study, we investigated the bone anabolic effects and calcium mobilizing potential of intermittent teriparatide (PTH (1–34)) and ABL using mole-to-mole matched doses (PTH (1–34): 100 µg/kg and ABL: 96 µg/kg) in mice.

## Material and Methods

### Animals and Grouping

In total, forty-seven female C57BL/6 mice with body weights of 21.1 ± 0.2 g were purchased from Janvier Labs (Le Genest-Saint-Isle, France). Upon arrival, the mice were stratified based on their body weight into four groups using custom-made software: Baseline (*n* = 11), Control (Ctrl) (*n* = 12), PTH (*n* = 12), and ABL (*n* = 12). At study start, the mice were 16 weeks old and had unrestricted access to tap water and standard pelleted mice chow (1324 maintenance diet for rats and mice, Altromin International, Lage, Germany) throughout the study. The mice were housed in standard plastic cages with 5–6 mice/cage and maintained at 21°C with a 12:12 hours light/dark cycle (6:00 am lights on/6:00 pm lights off). Mice were injected s.c. five days a week with either 100 µg/kg human PTH (1–34) (H-4835, Bachem, Bubendorf, Switzerland) or 96 µg/kg ABL (H-8334, Bachem, Bubendorf, Switzerland). Mice allocated to the Ctrl group were injected with saline in a similar way to serve as a control.

PTH has a relative molecular mass (M_r_) of 4117.77 Da and ABL has a M_r_ of 3960.64 Da, hence the dosing regimen used provided equal amounts in moles of either agent and thus allowed a direct mole-to-mole comparison of PTH and ABL.

The mice were injected with tetracycline (20 mg/kg, T3383, Sigma-Aldrich, St. Louis, MO, USA) four days before study start and alizarin (20 mg/kg, A3882, Sigma-Aldrich, St. Louis, MO, USA) four and eight days before sacrifice. The tetracycline label served as a baseline label, enabling estimation of bone erosion throughout the experiment as previously described (22). The Baseline group was sacrificed at study start, and the remaining groups were sacrificed after 21 days under anesthesia with 3% isoflurane (IsoFlo Vet, Orion Pharma Animal Health, Nivå, Denmark). One mouse in the Ctrl group was sacrificed before the end of the study because of failure to thrive.

All animal procedures were approved by the Danish Animal Inspectorate (2018-15-0201-01436).

### Tissue Extraction and Femoral Length

The right femora and tibiae and L4 vertebrae were isolated and any remaining soft tissue was carefully removed. Femora and L4s were stored in Ringer’s solution at −22°C, while tibiae were immersion-fixed in 0.1 M sodium phosphate-buffered formaldehyde (4% formaldehyde, pH 7.0) for 48 h and then stored in 70% ethanol. The femoral length was measured from the most distal part of the condyles to the top of the femoral head using a digital caliper.

### Dual-Energy X-Ray Absorptiometry (DXA)

Whole femora were scanned in a DXA scanner (Sabre XL, Norland Stratec, Pforzheim, Germany) at a pixel size of 0.1 mm × 0.1 mm and a scan speed of 3.0 mm/s as previously described (23) to determine the areal bone mineral density (aBMD) and bone mineral content (BMC). Quality assurance was performed by scanning solid-state phantoms in accordance with the manufacturer’s instructions.

### Micro Computed Tomography (μCT)

The femora and L4s were scanned in a μCT scanner (Scanco μCT 35, Scanco Medical AG, Brüttiselen, Switzerland) at an isotropic voxel size of 3.5 μm, an X-ray tube voltage of 55 kV, a current of 114 μA, with 1000 projections/180°, and an integration time of 800 ms. Beam hardening effects were reduced using a 0.5 mm aluminum filter.

**Figure 1:**
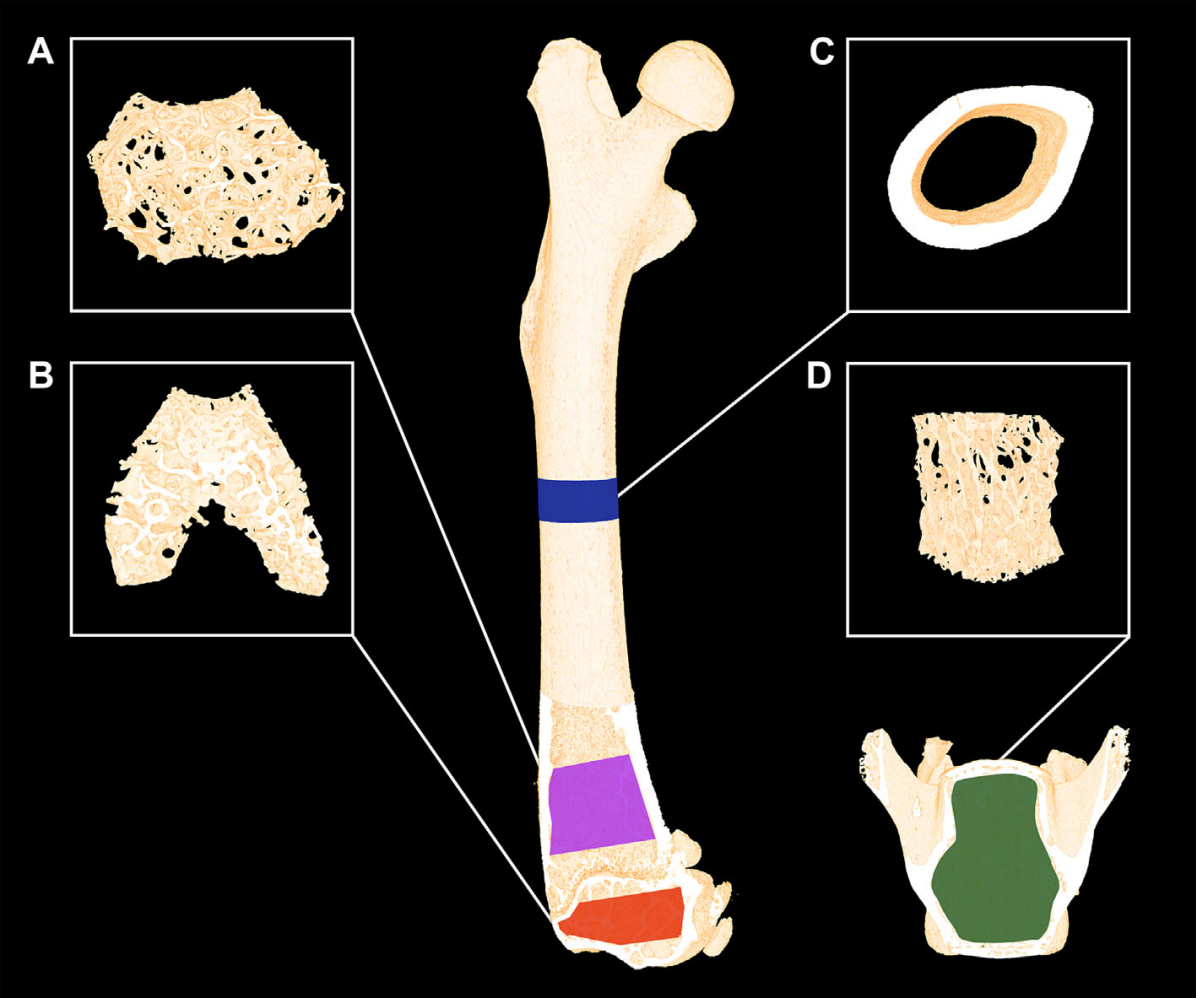
Micro-computed tomography (μCT) scan of a mouse femur and vertebra L4. **(A)** The magenta area represents the analyzed volume of interest (VOI) of the distal femoral metaphysis and **(B)** the yellow area represents the VOI of the distal femoral epiphysis consisting of trabecular bone only. **(C)** The blue areas represent the VOI consisting of cortical bone only at the femoral mid-diaphysis. **(D)** The green area represents the VOI containing trabecular bone only at vertebra L4. Dimensions are not to scale.

The femora were analyzed using separate volumes of interest (VOIs) at the mid-diaphysis, distal metaphysis, and distal epiphysis (**Figure 1**). The VOI at the femoral mid-diaphysis comprised a 0.82-mm-high region centered on the mid-point, thus containing cortical bone only. The VOI at the distal femoral metaphysis started 200 μm above the point, where the mineralized cartilage from the growth plate fused and ended 1000 μm further above and contained trabecular bone only. The femoral epiphyseal VOI started just after the medial and lateral epicondyle have fused to a coherent structure and ended, where the growth plate first appeared, thus containing trabecular bone only (**Figure 1**). The distal femoral metaphysis and epiphysis were both analyzed as site-specific differences between these skeletal sites have previously been reported (24). Finally, L4 was analyzed using a VOI spanning from the upper to the lower growth plate excluding primary spongiosa containing trabecular bone only.

The data were low-pass filtered using a Gaussian filter (σ = 0.8 and support = 1) and subsequently segmented with a fixed threshold filter determined automatically with IPL (v. 5.11, Scanco Medical AG, Switzerland) as previously described (23). The thresholds used were: femoral mid-diaphysis, distal metaphysis, and distal epiphysis: 527 mg HA/cm^3^ and L4: 529 mg HA/cm^3^.

The μCT analysis of cortical bone included: Ct.Th (cortical thickness), B.Ar (bone area), T.Ar (tissue area), and M.Ar (marrow area). The analysis of trabecular bone included: BV/TV (bone volume fraction), CD (connectivity density), Tb.N (trabecular number), Tb.Th (trabecular thickness), Tb.Sp (trabecular spacing), SMI (structure model index), vBMD (volumetric bone mineral density), and TMD (tissue mineral density). The assessment of bone microstructure was performed using the software supplied with the µCT scanner and followed the current guidelines (25).

### Mechanical Testing

Fracture strength and mechanical stiffness of the femoral mid-diaphysis, femoral neck, and L4 vertebral body were determined using a material testing machine (Instron model 5566, United Kingdom) as previously described in detail (23). Three bone sites were investigated since it has previously been reported that treatment with bone anabolic agents like intermittent PTH exhibit site-specificity of the mechanical properties (26).

### Dynamic Bone Histomorphometry

Dynamic bone histomorphometry was performed on the femoral mid-diaphysis and proximal tibial metaphysis to determine MS/BS (mineralizing surface/bone surface), MAR (mineral apposition rate), and BFR/BS (bone formation rate). Fluorescent labels (tetracycline and alizarin) were detected using a light microscope equipped with fluorescence light (Nikon Eclipse i80, Japan) and were quantified using newCAST (v. 2020.01, Visiopharm, Hørsholm, Denmark). If double labels were absent, an imputed value of zero was used to calculate MAR and BFR/BS. The distance between double labels was measured using the built-in measurement tool in newCAST, and fluorescent labels were quantified at a final magnification of ×1132. The assessment of bone histomorphometric measures and usage of related nomenclature was performed in accordance with the current recommendations by the ASBMR Histomorphometry Nomenclature Committee (27).

#### Cortical Bone

An approximately 200-μm-thick cross-sectional section was sawed with an Exakt saw (Apparatebau, Norderstedt, Germany) from the femoral mid-diaphysis. The unstained thick section was mounted on microscope slides and used to count fluorescent labels. A 24-arm grid was superimposed on the cortical bone surface so that the arms radiated from the center of the marrow cavity and intersected both the endocortical and periosteal bone surfaces. Only fluorescent labels on a bone surface intersecting the 24-arm grid were counted.

#### Trabecular Bone

7-μm-thick longitudinal sections of the proximal tibial metaphysis were cut on a microtome (Jung, RM2065; Leica Instruments, Nussloch, Germany) and left unstained. A 1000-μm-high region of interest (ROI) was delineated within the endocortical edges of the proximal tibial metaphysis starting 300 μm below the growth plate, thus excluding primary spongiosa and containing trabecular bone only. A random linear grid was superimposed on the live slide images in newCAST as previously described (28).

### Osteoid, Osteoblast, and Osteoclast-Covered Surfaces

The remaining 7-μm-thick longitudinal sections of the proximal tibial metaphysis were either stained with Masson-Goldner trichrome or stained for tartrate-resistant acid phosphatase (TRAP). The Masson-Goldner trichrome stained sections were used to determine OS/BS (osteoid-covered surfaces) and Ob.S/BS (osteoblast-covered surfaces). Osteoid was defined as a bright red unmineralized region at the very edge of the trabeculae, and osteoblasts were defined as mononucleated cuboidal cells residing at the bone surface.

The sections stained for TRAP were used to determine Oc.S/BS (osteoclast-covered surfaces). Osteoclasts were defined as TRAP-positive multinucleated cells residing at the bone surface. The Masson-Goldner trichrome and TRAP-stained sections were analyzed using the same ROIs as used for dynamic bone histomorphometry of the proximal tibial metaphysis. The analysis of OS/BS, Ob.S/BS, and Oc.S/BS were performed at a final magnification of ×1132.

### Serum Biochemistry

When the mice were sacrificed, blood was collected from the inferior vena cava and stored in single-use plastic tubes on ice for 15 minutes. Serum was separated by centrifugation at 1100 g for 10 minutes at 4°C and stored at −80°C for the subsequent analysis. Calcium ion content in the serum was determined using a commercially available colorimetric assay kit (MAK022, Sigma-Aldrich, St. Louis, MO, USA) in accordance with the manufacture’s guidelines. Calcium ion content in the samples were determined using a standard curve generated by measuring a series of samples with known concentrations of ionized calcium. All samples were analyzed in duplicates.

### Statistics

All animals were investigated with all of the above described methods. Data were analyzed using a one-way parametric ANOVA followed by a post-hoc Holm-Sidak test, whenever normal distributions requirements were met. If the data were not normally distributed, a non-parametric one-way ANOVA on ranks was performed followed by a post-hoc Dunn’s test instead. In either case, the ANOVA analysis did not include the Baseline group. Normality was assessed using Q–Q plots and the D’Agostino-Pearson normality test. Results were defined as statistically significant if the *p*-values were below 0.05. All statistical analyses were performed in GraphPad Prism 8.4.0 (GraphPad Software, San Diego, CA, USA). An *a priori* sample size calculation (power = 0.8) on C57/BL6 mice showed that a 5% difference in femoral aBMD and a 15% difference in mid-diaphyseal bone strength can be demonstrated between groups with *n* = 12 animals.

## Results

### Animals and DXA

The body weights and femoral lengths did not differ significantly between groups at either initiation or termination of the study. Treatment with ABL significantly increased aBMD (+6%) compared to Ctrl, while treatment with PTH resulted in a borderline significant (*p* = 0.06) increase of aBMD by 4%. BMC did not differ in animals treated with either PTH or ABL and Ctrl animals after 21 days of treatment. Neither aBMD nor BMC differed between the PTH- and ABL-treated animals (**Table 1**).

**Table 1:** Initial and final body weight, femoral length, and BMC of mice treated with PTH or ABL for 21 days.

	Baseline	Ctrl	PTH	ABL
Body weight (g) (study start)	20.8 ± 1.5	21. 2 ± 0.9	21. 2 ± 0.9	21.3 ± 1.3
Body weight (g) (study end)	20.8 ± 1.5	21.9 ± 1.1	22.7 ± 1.0	22.9 ± 1.5
Femoral length (mm)	15.3 ± 0.3	15.8 ± 0.3	15.7 ± 0.3	15.7 ± 0.3
aBMD (mg/cm^2^)	57.0 ± 1.9	63.6 ± 2.2	66.2 ± 3.4	67.1 ± 3.6^*^
BMC (mg)	19.6 ± 1.2	22.8 ± 1.5	24.0 ± 1.5	23.8 ± 2.0

aBMD, areal bone mineral density; BMC, bone mineral content. Data are presented as mean ± SD.

### μCT

#### Femoral Mid-Diaphysis

Treatment with ABL significantly increased femoral mid-diaphyseal Ct.Th (+6%) and B.Ar (+9%) compared to Ctrl, while treatment with PTH did not. T.Ar and M.Ar did not differ between any of the groups. Neither of the investigated cortical bone parameter differed between the PTH- and ABL-treated animals (**Table 2**).

**Table 2:** Femoral and vertebral bone structural parameters determined by μCT of mice treated with PTH or ABL for 21 days.

	Baseline	Ctrl	PTH	ABL
**Mid-diaphysis**				
Ct.Th (μm)	166 ± 5.6	180 ± 6.1	185 ± 5.6	190 ± 5.5^*^
B.Ar (mm^2^)	0.71 ± 0.04	0.80 ± 0.04	0.83 ± 0.03	0.87 ± 0.04^*^
T.Ar (mm^2^)	1.66 ± 0.10	1.71 ± 0.07	1.73 ± 0.05	1.73 ± 0.07
M.Ar (mm^2^)	0. 90 ± 0.07	0.90 ± 0.05	0.89 ± 0.04	0.87 ± 0.06
**Distal metaphysis**				
Tb.Th (μm)	31.1 ± 2.1	36.7 ± 1.8	38.2 ± 3.6	36.9 ± 3.9
Tb.N (mm^−1^)	4.0 ± 0.1	3.7 ± 0.2	3.9 ± 0.2	4.1 ± 0.5
Tb.Sp (μm)	249 ± 9.5	270 ± 11.2	268 ± 14.5	260 ± 27.2
CD (mm^−3^)	276 ± 96.1	284 ± 82.8	694 ± 266^*^	886 ± 362^*,#^
SMI (–)	2.71 ± 0.21	1.88 ± 0.26	1.35 ± 0.16^*^	1.28 ± 0.32^*^
vBMD (mg/cm^3^)	91.1 ± 10.5	103 ± 16.8	134 ± 19.5^*^	146 ± 38.2^*^
TMD (mg/cm^3^)	919 ± 9.3	931 ± 7.7	919 ± 9.9^*^	915 ± 16.0^*^
**Distal epiphysis**				
Tb.Th (μm)	52.2 ± 0.7	65.6 ± 0.4	67.9 ± 0.4	67.1 ± 0.6
Tb.N (mm^−1^)	5.40 ± 0.53	5.71 ± 0.41	6.38 ± 0.39^*^	6.54 ± 0.59^*^
Tb.Sp (μm)	209 ± 18.1	207 ± 14.0	197 ± 12.2	191 ± 17.7^*^
CD (mm^−3^)	352 ± 69.0	310 ± 73.9	395 ± 73.0^*^	419 ± 96.7^*^
SMI (–)	−0.08 ± 0.80	−0.62 ± 0.19	− 1.10 ± 0.24^*^	−1.10 ± 0.34^*^
vBMD (mg/cm^3^)	310 ± 77.9	387 ± 27.8	424 ± 19.9^*^	420 ± 32.7^*^
TMD (mg/cm^3^)	999 ± 27.4	1037 ± 5.8	1027 ± 5.7^*^	1021 ± 12.4^*^
**Vertebra (L4)**				
BV/TV (%)	17.1 ± 1.43	17.2 ± 1.08	20.3 ± 1.5^*^	21.0 ± 1.97^*^
Tb.Th (μm)	34.9 ± 1.39	35.4 ± 1.57	34.3 ± 1.48	34.2 ± 0.97
Tb.N (mm^−1^)	4.66 ± 0.29	4.52 ± 0.22	5.21 ± 0.36^*^	5.45 ± 0.55^*^
Tb.Sp (μm)	223 ± 13.4	231 ± 12.0	206 ± 15.2^*^	198 ± 18.9^*^
CD (mm^−3^)	459 ± 55.4	460 ± 44.8	996 ± 77.7^*^	1115 ± 156^*,#^
SMI (–)	0.45 ± 0.09	0.36 ± 0.09	0.33 ± 0.13	0.38 ± 0.08
vBMD (mg/cm^3^)	188 ± 16.1	187 ± 20.3	221 ± 16.2^*^	226 ± 22.9^*^
TMD (mg/cm^3^)	914 ± 6.43	923 ± 6.69	899 ± 7.73^*^	900 ± 5.85^*^

Data are presented as mean ± SD. *p < 0.05 vs. Ctrl. ^#^p < 0.05 vs. PTH; Ct.Th, cortical thickness; B.Ar, bone area; T.Ar, diaphyseal tissue area; M.Ar, marrow area; Ct.Po, cortical porosity; Tb.Th, trabecular thickness; Tb.N, trabecular number; Tb.Sp, trabecular spacing; CD, connectivity density; SMI, structure model index; vBMD, volumetric bone mineral density; TMD, tissue mineral density.

#### Distal Femoral Metaphysis

**Figure 2:**
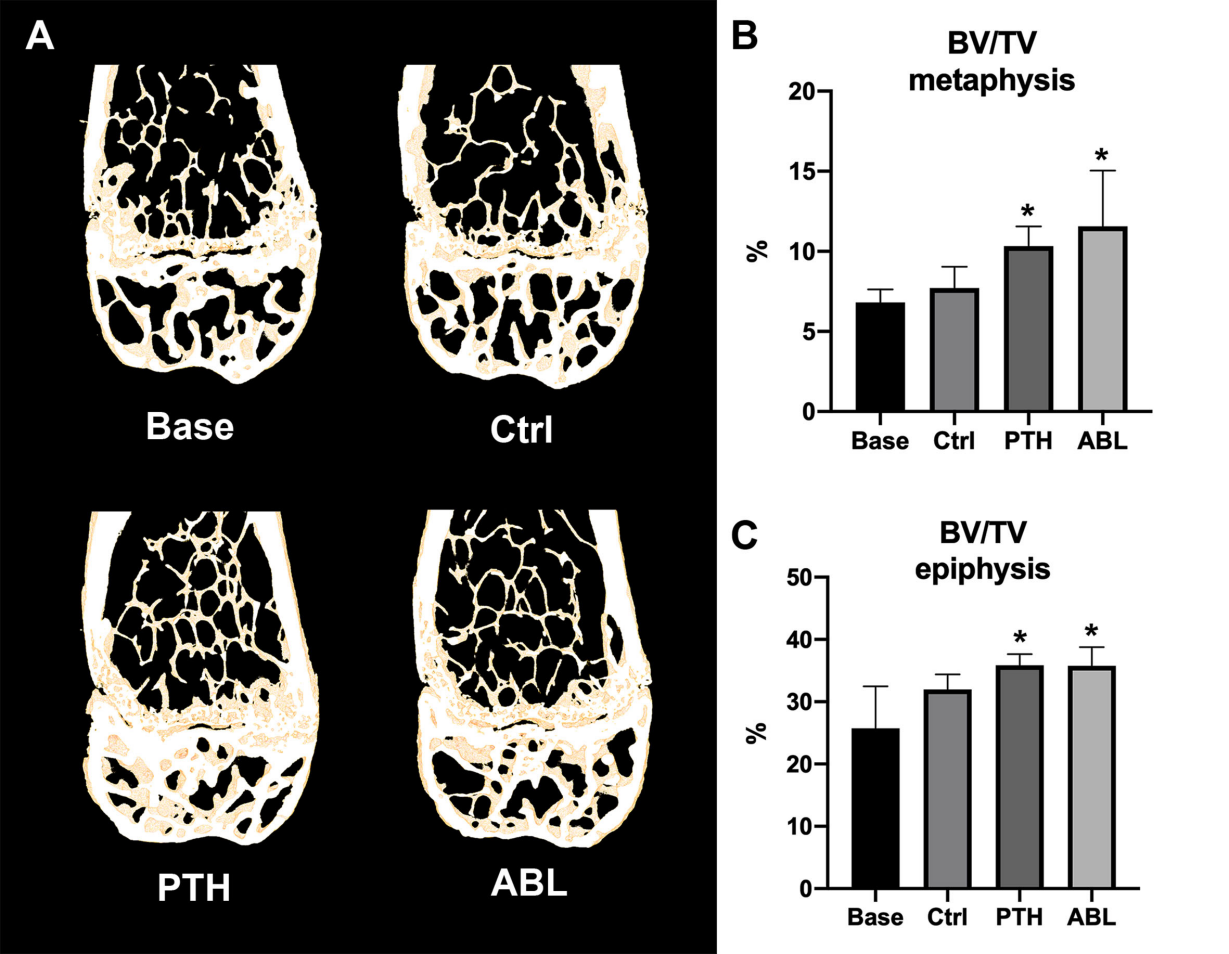
**(A)** 3D reconstructions of 105-μm-thick representative frontal slices through the distal femoral metaphyseal and epiphyseal. **(B, C)** Bone volume fraction (BV/TV) at the distal femoral metaphysis and epiphysis determined by μCT. Data are presented as mean ± SD. *p < 0.05 vs. Ctrl.

Treatment with either PTH or ABL significantly increased trabecular BV/TV (+37% and +54%), CD (+144% and +212%), and vBMD (+30% and +42%) compared to Ctrl, respectively (**Table 2** and **Figure 2**). In addition, both TMD (−1% and −2%) and SMI (−28% and −32%) significantly decreased after 21 days of treatment with PTH or ABL compared to Ctrl. The metaphyseal bone gain manifested itself as a decrease in SMI indicating a change in trabecular geometry from a more rod-like toward a more plate-like structure. CD differed significantly between PTH and ABL, where treatment with ABL resulted in 28% higher CD compared to treatment with PTH, while neither of the other microstructural parameters differed between animals treated with PTH and ABL (**Table 2** and **Figure 2**).

#### Distal Femoral Epiphysis

The trabecular microarchitectural changes were less profound at the distal femoral epiphysis than at the distal femoral metaphysis. Thus, treatment with PTH significantly increased BV/TV (+12%), Tb.N (+12%), CD (+27%), and vBMD (+10%) and decreased TMD (−1%) and SMI (−77%) compared to Ctrl. Likewise, treatment with ABL significantly increased BV/TV (+12%), Tb.N (+15%), CD (+35%), and vBMD (+9%) compared to Ctrl. In addition to a significant decrease in TMD (−2%) and SMI (−77%), treatment with ABL also significantly decreased Tb.Sp (−8%) compared to Ctrl. Treatment with PTH or ABL did not have a significant effect on either Tb.Th or the remaining microstructural parameters (**Table 2** and **Figure 2**). Neither of the investigated microstructural parameter at the distal femoral metaphysis differed between the PTH- and ABL-treated animals (**Table 2**).

#### Vertebra L4

Treatment with either PTH or ABL significantly increased trabecular BV/TV (+18% and +22%), Tb.N (+15% and +21%), CD (+117% and +143%), and vBMD (+18% and +21%) compared to Ctrl, respectively. Furthermore, these trabecular changes were accompanied by significant reductions in Tb.Sp (−11% and −14%) and TMD (−3% and −2%) compared to Ctrl, respectively. None of the spinal trabecular bone parameters differed between PTH- and ABL-treated animals except for CD, which was larger (+12%) in ABL-treated animals that in PTH-treated animals (**Table 2**).

### Mechanical Testing

Treatment with either PTH or ABL significantly increased mechanical strength (+16% and +23%) and stiffness (+17% and +21%) at the femoral mid-diaphysis compared to Ctrl, respectively. No significant difference was found in either bone strength or stiffness at the femoral neck or L4 vertebral body between any of the groups (**Table 3**).

**Table 3:** Mechanical properties of the femoral mid-diaphysis, femoral neck, and vertebral body of L4 from mice treated with PTH or ABL for 21 days.

	Baseline	Ctrl	PTH	ABL
**Mid-diaphysis**				
Strength (N)	12.8 ± 1.30	15.0 ± 2.58	17.4 ± 1.05^*^	18.5 ± 1.66^*^
Stiffness (N/mm)	94.6 ± 11.6	106 ± 16.3	124 ± 10.1^*^	128 ± 14.5^*^
**Neck**				
Strength (N)	10.3 ± 1.65	12.0 ± 1.67	13.3 ± 1.46	13.6 ± 1.89
Stiffness (N/mm)	77.4 ± 10.6	100 ± 27.1	113 ± 30.2	96.8 ± 13.62
**L4**				
Strength (N)	17.6 ± 3.39	19.0 ± 4.60	19.9 ± 2.72	21.0 ± 5.62
Stiffness (N/mm)	144 ± 29.9	189 ± 70.6	169 ± 41.7	178 ± 41.4

Data are presented as mean ± SD. *p < 0.05 vs. Ctrl.

### Dynamic Bone Histomorphometry

#### Femoral Mid-Diaphysis

Treatment with PTH significantly increased periosteal MS/BS (+43%), MAR (+690%), and BFR/BS (+540%), whereas treatment with ABL significantly increased MS/BS (+63%) and BFR/BS (+560%), but not MAR. No significant differences were observed at the endocortical envelope between any of the groups. In addition, the effect of PTH and ABL on dynamic histomorphometry parameters did not differ significantly at either the periosteal or endocortical envelope at the femoral mid-diaphysis (**Table 4**).

**Table 4:** Dynamic bone histomorphometry at the femoral endocortical (Ec) and periosteal (Ps) mid-diaphysis and at the proximal tibial metaphysis (trabecular only).

	Baseline	Ctrl	PTH	ABL
**Endocortical**				
Ec.Tetra.S/BS (%)	87.1 ± 5.97	66.0 ± 11.2	66.4 ± 8.18	71.5 ± 10.2
Ec.MS/BS (%)	(–)	40.7 ± 5.51	45.1 ± 8.14	42.2 ± 15.4
Ec.MAR (μm/d)	(–)	0.42 ± 0.43	0.76 ± 0.58	0.67 ± 0.54
Ec.BFR/BS (μm^3^/μm^2^/d)	(–)	0.17 ± 0.19	0.33 ± 0.24	0.32 ± 0.27
**Periosteal**				
Ps.Tetra.S/BS (%)	36.1 ± 11.5	43.0 ± 14.6	50.7 ± 14.9	57.4 ± 13.6
Ps.MS/BS (%)	(–)	27.1 ± 9.0	38.9 ± 7.70*	44.2 ± 15.4^*^
Ps.MAR (μm/d)	(–)	0.10 ± 0.32	0.79 ± 0.69^*^	0.66 ± 0.66
Ps.BFR/BS (μm^3^/μm^2^/d)	(–)	0.05 ± 0.15	0.32 ± 0.28^*^	0.33 ± 0.30^*^
**Trabecular**				
Tetra.S/BS (%)	58.8 ± 8.54	14.5 ± 6.72	8.36 ± 4.84	8.10 ± 5.41
MS/BS (%)	(–)	46.4 ± 5.10	46.8 ± 5.65	48.0 ± 6.64
MAR (μm/d)	(–)	1.85 ± 0.37	2.39 ± 0.36^*^	2.88 ± 0.25^*^
BFR/BS (μm^3^/μm^2^/d)	(–)	0.86 ± 0.19	1.11 ± 0.15^*^	1.10 ± 0.18^*^

Mice were s.c. injected with tetracycline four days before study start and alizarin four and eight days before sacrifice. Tetra.S/BS, tetracycline-covered bone surfaces; MS/BS, mineralizing surface/bone surface; MAR, mineral apposition rate; BFR/BS, bone formation rate; Data are presented as mean ± SD. *p < 0.05 vs. Ctrl. Graphs illustrating the data as box and whisker plots and scatter plots are available in the **Supplementary Material** (**Figure S1**).

#### Proximal Tibial Metaphysis

Treatment with either PTH or ABL significantly increased MAR (+29% and +56%) and BFR/BS (+29% and +28%) compared to Ctrl, while neither treatment had any significant effect on MS/BS. In addition, Tetra.S/BS was significant lower in mice treated with PTH (−42%) or ABL (−44%) compared to non-treated Ctrl mice, indicating increased bone resorption in the treated groups. The effect of PTH and ABL on the dynamic histomorphometry parameters did not differ at the proximal tibial metaphysis (**Table 4**).

### Osteoid, Osteoblast, and Osteoclast-Covered Surfaces

Treatment with PTH or ABL had no significant effect on either OS/BS, Ob.S/BS, or Oc.S/BS after 21 days (**Table 5**). However, a trend toward increased Ob.S/BS was observed in mice treated with either PTH or ABL compared to Ctrl.

**Table 5:** Assessment of trabecular bone formation and bone resorption at the proximal tibial metaphysis.

	Baseline	Ctrl	PTH	ABL
OS/BS (%)	29.9 ± 7.64	26.6 ± 7.92	26.3 ± 8.87	23.3 ± 9.46
Ob.S/BS (%)	9.75 ± 7.33	9.11 ± 5.61	18.9 ± 11.2	15.6 ± 10.8
Oc.S/BS (%)	21.5 ± 3.85	22.2 ± 7.54	26.4 ± 4.97	24.9 ± 8.61

OS/BS, osteoid-covered surfaces; Ob.S/BS, osteoblast-covered surfaces; Oc.S/BS, osteoclast-covered surfaces. Data are presented as mean ± SD. Graphs illustrating the data as box and whisker plots and scatter plots are available in the **Supplementary Material** (**Figure S2**).

### Serum Biochemistry

No significant difference in serum calcium was found between any of the groups (**Table 6**).

**Table 6:** Ionized serum calcium levels from animals treated with PTH or ABL.

	Baseline	Ctrl	PTH	ABL
Serum Ca^2+^ (mmol/l)	2.31 ± 0.20	2.44 ± 0.18	2.33 ± 0.13	2.39 ± 0.08

Data are presented as mean ± SD.

## Discussion

The purpose of the present study was to compare the bone anabolic efficacy of PTH and ABL in a direct mole-to-mole fashion in mice. The main finding of the study was that treatment with PTH or ABL has similar effects on bone in mice.

In contrast to the recent study by Le Henaff et al., we found that Tb.Th was not significantly increased at any of the three trabecular bone sites investigated with μCT. Instead we found significantly higher Tb.N at the distal femoral epiphysis and L4 in mice treated with PTH or ABL compared to control mice. The observed increase in BV/TV and vBMD could therefore be explained by the higher Tb.N and not by the increased Tb.Th as reported by Le Henaff et al. (21). Furthermore, we found that treatment with PTH or ABL significantly decreased TMD at both the distal femoral metaphysis and epiphysis and L4, reflecting that newly formed bone is less mineralized than mature bone. The differences between the outcome of the present study and that of the study by Le Henaff et al. might be explained, at least partly, by differences in study duration, sex of mice, and μCT voxel size. The study by Le Henaff et al. used male mice, a study duration of six weeks, and a voxel size of 9.7 μm for their μCT analysis (21), while we used female mice, a shorter study duration of three weeks, and a substantially smaller voxel size of 3.5 μm.

In the present study, we found that ABL and PTH significantly increased the mid-diaphyseal bone strength accompanied by an increase in mid-diaphyseal Ct.Th and B.Ar, although this was statistically significant for ABL-treated mice only. The increased Ct.Th after treatment with ABL is also in accordance with the mid-diaphyseal μCT findings reported by Le Henaff et al. (21) and mechanical testing from ABL-treated ovariectomized rats reported by Varela et al. (29).

Le Henaff et al. were surprised by the similar effect of ABL and PTH because preclinical and clinical findings have reported conflicting results (21). The present study confirms their observation that ABL and PTH have similar effects on murine bone. Moreover, recently we established that PTH and ABL were similar effective in counteracting disuse osteopenia in rats when given in mole-to-mole comparable doses (30).

The bone anabolic effect of PTH and ABL materialized without a significant increase in osteoblast-covered surfaces. This might suggest that recruitment of osteoblasts, from either osteoblast progenitor cells (12) or bone lining cells (31), occurred early in the experiment and had leveled out when the mice were sacrificed. However, it is worth mentioning that we found a trend toward increased osteoblast-covered surfaces in the PTH- and ABL-treated mice with a large within-group variation, which may explain why the increase in osteoblast covered surfaces was not statistically significant.

Several preclinical studies have compared the effect of multiple concentrations of ABL to determine optimal dosing (32–34), while only few preclinical studies have compared the effect of PTH and ABL (20, 21). The study by Bernhardsson et al. showed that PTH and ABL can stimulate bone regeneration in a murine fracture model to a similar extent, however, the study did not use comparable doses of PTH and ABL (20).

The large multicenter double-blinded randomized clinical phase 3 trial conducted by Miller et al. compared the effect of PTH and ABL in post-menopausal osteoporotic women. The study showed that the increase in total hip BMD was greater in the ABL-treated group than in the PTH-treated group. However, it should be emphasized that even though the clinical effect of ABL might be similar or better compared to PTH in that study, the dose of ABL (80 μg) was four times higher than of the dose of PTH (20 μg) (18). Despite the four times higher dose of ABL they nevertheless found that hypercalcemia was less frequent in patients treated with ABL than in patients treated with teriparatide (18). This is consistent with ABL being exalted for its ability to increase bone formation and bone mineral density with less calcium-mobilizing potential than teriparatide (35). Interestingly, the observation of the present study, that ABL and PTH results in similar calcium concentrations in mice is somewhat conflicting with the clinical findings by Miller et al. (18). However, we and others have previously reported no differences in either serum calcium levels in rats (30) and mice (36, 37) or CTX-I levels (21, 38) in mice treated with ABL or PTH in comparable doses, indicating that PTH and ABL have similar calcium mobilizing effects in rodents. The difference in hypercalcemia in preclinical studies in rodents and clinical studies might be attributed to species differences.

A limitation of the present study is the relatively high dose of PTH compared to the dose used in a clinical setting. However, the dose of PTH was based on previous studies in mice (39–41) in order to maximize the bone anabolic effect and the dose of ABL was adjusted accordingly to enable a direct mole-to-mole comparison of the two anabolic treatment regimens. Another limitation is it that the study was conducted in intact mice and therefore the findings are valid for non-osteopenic animals only.

In conclusion, abaloparatide and PTH are in general similar effective as osteoanabolic agents to promote bone mineral density, trabecular microarchitecture, and bone strength in mice.

## Data Availability Statement

The raw data supporting the conclusions of this article will be made available by the authors, without undue reservation.

## Ethics Statement

The animal study was reviewed and approved by Danish Animal Inspectorate (2018-15-0201-01436).

## Author Contributions

Study design: MB, AB, and JT. Study conduct: MB and AB. Data collection, data analysis, and interpretation: MB, FS, AB, and JT. Manuscript draft: MB. Figures and graphical design: MB. Manuscript revision: MB, AB, and JT. All authors contributed to the article and approved the submitted version.

## Conflict of Interest

The authors declare that the research was conducted in the absence of any commercial or financial relationships that could be constructed as a potential conflict of interest.
