# Supplementary material for: Teriparatide and Abaloparatide Have a Similar Effect on Bone in Mice
Source: Front Endocrinol (Lausanne). 2021 Apr 19;12:628994. doi: 10.3389/fendo.2021.628994 (PMC8092394; doi:10.3389/fendo.2021.628994)

Figure S1. Box and whisker plots of mid-diaphyseal femoral periosteal (Ps) and endocortical (Ec) (A) mineralizing surfaces (MS/BS), (B) mineral apposition rate (MAR), and (C) bone formation rate (BFR/BS) from mice treated with teriparatide (PTH) or abaloparatide (ABL) for 21 days. Individual data points are shown as filled circles, the median is shown as a vertical line, the box represent the Q1 and Q3 interquartile range, and the whiskers shows minimum and maximum values in the date set.

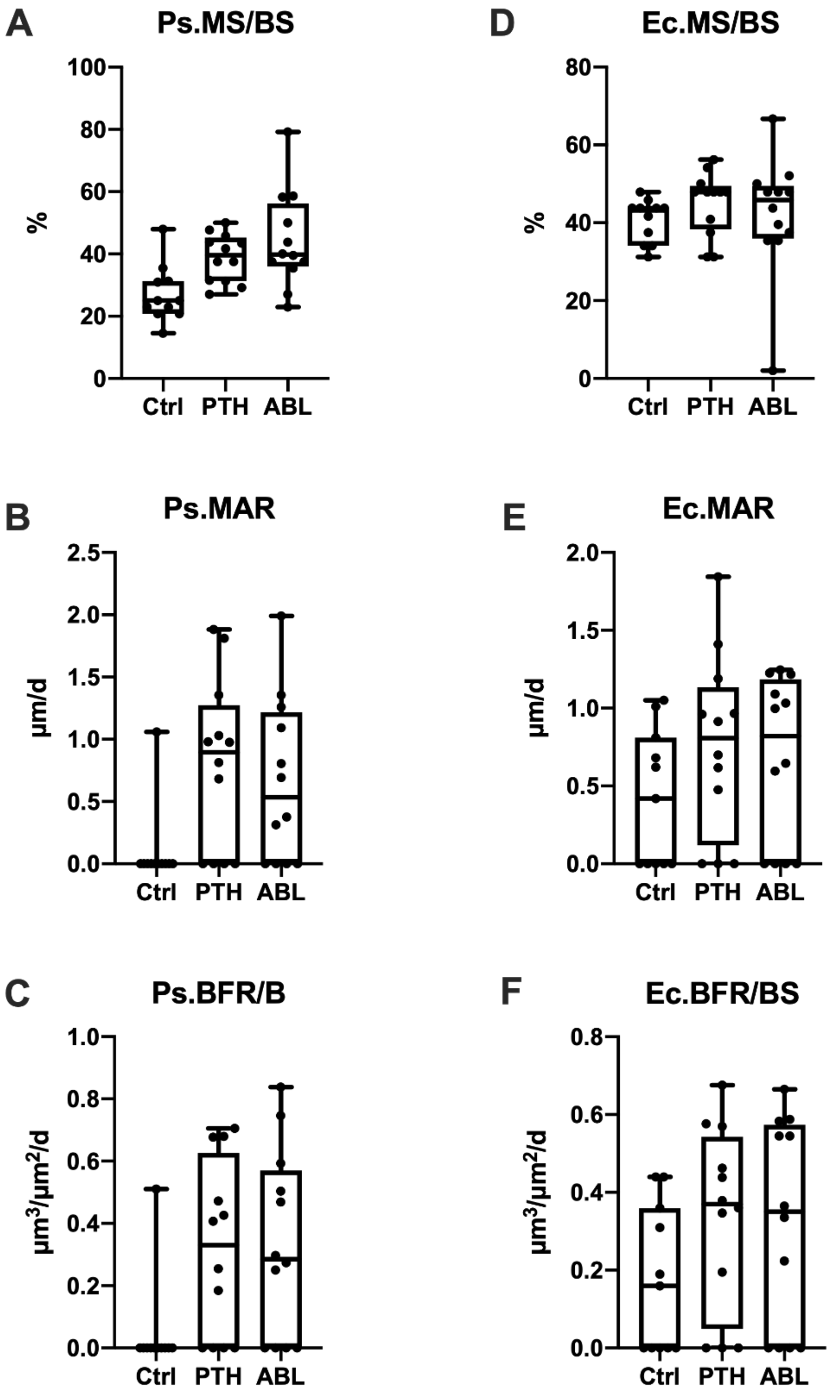

Figure S2. Box and whisker plots of femoral metaphyseal trabecular (A) mineralizing surfaces (MS/BS), (B) mineral apposition rate (MAR), (C) bone formation rate (BFR/BS), (D) osteoid-covered surfaces (OB/BS), (E) osteoblast-covered surfaces (Ob.S/BS), and (F) osteoclast-covered surfaces (Oc.S/BS) from mice treated with teriparatide (PTH) or abaloparatide (ABL) for 21 days. Individual data points are shown as filled circles, the median is shown as a vertical line, the box represent the Q1 and Q3 interquartile range, and the whiskers shows minimum and maximum values in the date set.

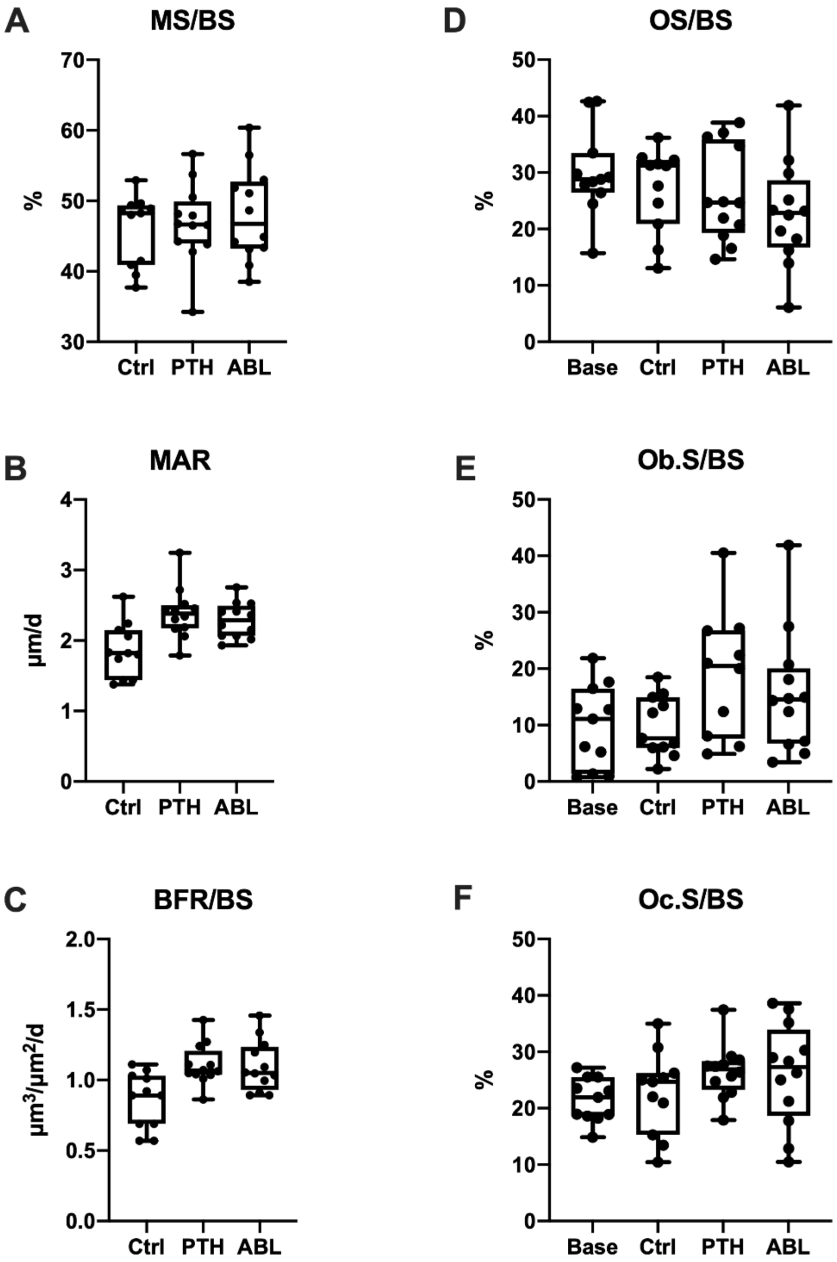

Supplement: Supplementary file 1 [file DataSheet_1.pdf]
